# Nanocrystals and nanosuspensions: an exploration from classic formulations to advanced drug delivery systems

**DOI:** 10.1007/s13346-024-01559-0

**Published:** 2024-03-07

**Authors:** Benjamin Rossier, Olivier Jordan, Eric Allémann, Carlos Rodríguez-Nogales

**Affiliations:** 1 School of Pharmaceutical Sciences, University of Geneva, Rue Michel-Servet 1, 1211 Geneva 4, Switzerland; 2 Institute of Pharmaceutical Sciences of Western Switzerland, Rue Michel-Servet 1, 1211 Geneva 4, Switzerland

**Keywords:** Nanocrystals, Nanosuspension, Poorly soluble drugs, Drug delivery systems, Itraconazole

## Abstract

Nanocrystals and nanosuspensions have become realistic approaches to overcome the formulation challenges of poorly water-soluble drugs. They also represent a less-known but versatile platform for multiple therapeutic applications. They can be integrated into a broad spectrum of drug delivery systems including tablets, hydrogels, microneedles, microparticles, or even functionalized liposomes. The recent progresses, challenges, and opportunities in this field are gathered originally together with an informative case study concerning an itraconazole nanosuspension-in-hydrogel formulation. The translational aspects, historical and current clinical perspectives are also critically reviewed here to shed light on the incoming generation of nanocrystal formulations.

## Graphical abstract

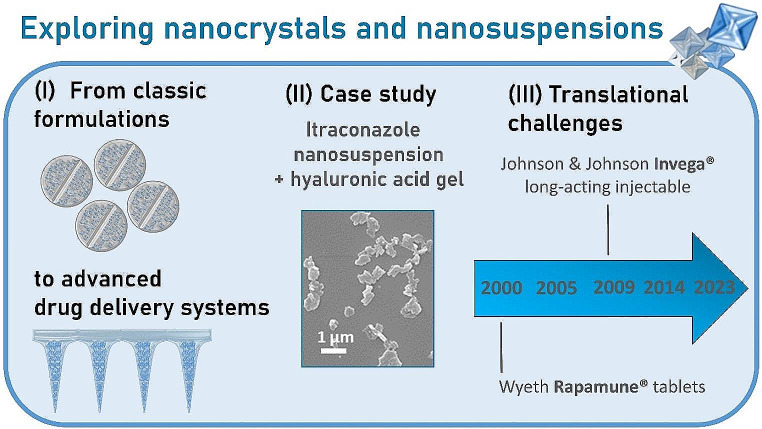

## Introduction

An increasing number of new drug candidates are characterized by an extremely low solubility, belonging to class II or class IV of the Biopharmaceutics Classification System (BCS) [1, 2]. This dramatically hampers their drugability unless they enter into a tedious reformulation process. The significant progress of nanotechnology has also provided new opportunities for the task over classic solubilization strategies such as salt formation, co-crystal formation, or cyclodextrin complexation. Among the different applications in the field, the conceptualization of nanocrystals (i.e., a crystalline drug with a particle size below the micrometer scale) and/or nanosuspensions (i.e., crystalline, semi-crystalline or amorphous drug aqueous nanosuspensions) has become a feasible approach to overcome the formulation challenges of poorly water-soluble drugs [3, 4].

**Fig. 1:**
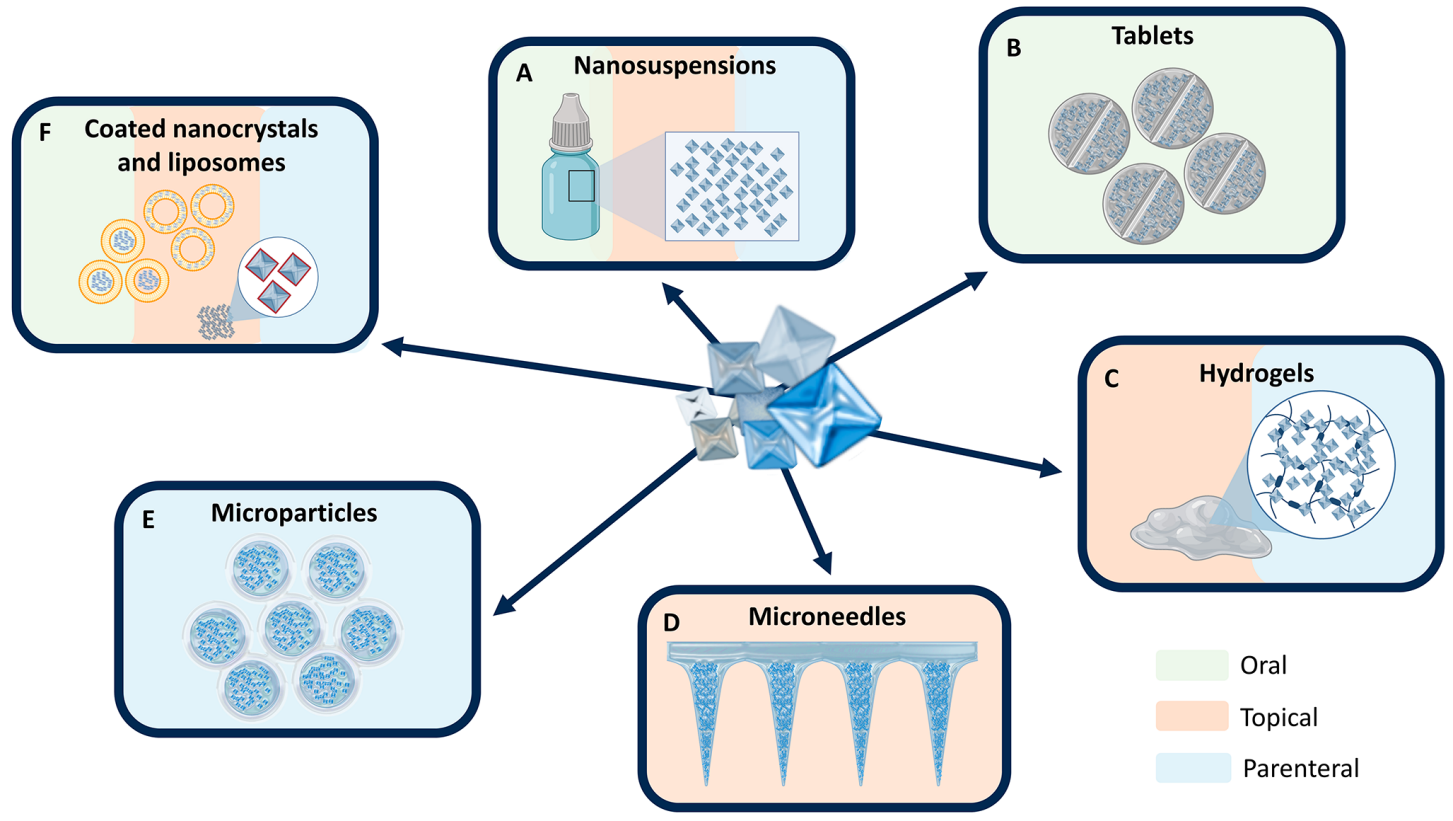
Schematic representation of formulations and drug delivery systems recently proposed for drug nanocrystals and nanosuspensions matched with their corresponding administration routes (see color code). The topical route includes ocular delivery. Nanosuspensions have been implemented in the past for pulmonary drug delivery. (A) Nanosuspensions, (B) tablets, (C) hydrogels, (D) microneedles, (E) microparticles, and (F) coated nanocrystals and liposomes. (Designed by Biorender)

Nanocrystals and nanosuspensions are crystalline or partly amorphous nano-sized structures of active compounds obtained through two main methods: one involves size reduction of larger crystals mainly by milling or high-pressure homogenization techniques (known as the top-down approach), whereas the other involves precipitation of dissolved molecules mainly by crystallization or spray-drying (referred to as the bottom-up approach) [5, 6]. One of their significant and well-known advantages is their high specific surface area. According to the Noyes-Whitney equation, this favors a faster dissolution; and additionally an increased saturation concentration (Freundlich-Ostwald relation) [7, 8]. This, together with a very high drug loading, and a nanosized particle distribution has promoted their implementation in formulation development [4, 9]. Taking advantage of these assets, formulation scientists have been mainly focused on improving the oral bioavailability of poorly water-soluble drugs over the last two decades [6, 9–11]. Literature inputs various reports on this application with particular emphasis on the formulation methodology and solid-state characterization of nanocrystals (see for instance the following review articles [6, 12, 13]). However, there exists nowadays a growing interest in exploring other opportunities. This not only entails other administration routes but also a search for alternative strategies to improve current treatments lacking efficacy. Nanocrystals or nanosuspensions represent in fact a less known but versatile platform for multiple applications. They possess a high particle engineering potential and can be linked to a broad spectrum of drug delivery systems (Fig. 1).

This review article aims to provide an up-to-date, comprehensive, and cross-disciplinary overview of some of the latest improvements in the field of nanocrystal technology for different applications. Particular attention is given for the first time to their integration in different drug delivery systems, ranging from classic nanosuspensions or tablet formulations to novel microneedles or targeted nanocrystals. In order to illustrate further the potential of these approaches, and the technicalities, we have included an informative and original case study dealing with the formulation and characterization of a nanosuspension-hydrogel of the poorly water-soluble drug itraconazole. To conclude, the historical and current perspectives in the clinic are addressed. The section contains a critical view with relevant information about the challenges and opportunities of these technology approaches. All the insights gathered will help researchers, formulators, and clinicians to comprehend better their current translational reality.

## Literature data collection and analysis

### Literature review

For the selection of publications presented in Sect. 3, a MEDLINE Boolean literature search was mainly conducted using the keywords “nanocrystals” OR “nanomilling” AND “application” AND “formulation”. Papers and reviews with the mentioned contents and published over the last 5 years were included in the review process. Only the most relevant studies and majorly containing in vivo studies are highlighted in the text.

Clinical trials research in Sect. 5 included studies exclusively on drug nanocrystals and nanosuspensions. Data was extracted from the database ‘clinicaltrials.gov’, powered by the United States National Library of Medicine. In Sect. 5, Fig. 4F, the total number of publications per year was extracted from MEDLINE using the following Boolean search query: “nanocrystal” OR “nanosuspension” AND “drug” NOT “nanoparticle”.

### Formulation and characterization of an itraconazole nanosuspension

Size reduction was performed using a homogenizer (Precellys 24^®^) from Bertin Instruments (Montigny-le-Bretonneux, France). Itraconazole (Parchem, New Rochelle, NY, USA) was added to a 2-mL tube with the stabilizer poloxamer 407 (Sigma-Aldrich, Saint Louis, MO, USA) at a drug-to-stabilizer w/w ratio 1/0.1. Then, 1 g of 0.5-mm zirconium oxide beads (Next Advance, Troy, NY, USA) and 1 mL of Milli-Q water were added. The complete sequence consisted of 50 cycles of 70 s at 6,600 rpm. The obtained nanosuspension was also loaded in a preformed hydrogel containing 1.9 MDa hyaluronic acid (Bloomage Biotech, Jinan, China) at 2% w/v. Briefly, the aqueous nanosuspension was added to the gel and mixed gently until a homogeneous blend was obtained at the final drug concentration of 0.5 mg/mL.

Particle size distribution by specific surface density was determined using a Mastersizer 3000 (Malvern, Worcestershire, UK) laser diffraction equipment. Particle population distribution by intensity and mean particle size diameter expressed as mean Z-average was determined by dynamic light scattering (Zetasizer Nano ZS Malvern; Malvern Instruments SA, UK). The refractive index was set at 1.33 and the scattering angle was 173°. Particles’ morphology was visualized using a JSM-7001 F emission scanning electron microscope (SEM) (JEOL, Tokyo, Japan). Samples were previously sputter-coated with gold and all micrographs were acquired at a 15 kV voltage.

In vitro dissolution studies were performed in a HCl (Acros Organics, Geel, Belgium) 0.1 N solution to increase the solubility of itraconazole. Suspensions were incubated at a concentration of 10 µg/mL in an orbital shaker at 80 rpm at 37 °C. At allotted time points, each tube was centrifuged at 4,000 rpm at 37 °C for 5 min to recover supernatants. Samples were centrifuged at 10,000 rpm at 37 °C for 10 min and then filtered using 0.22 μm centrifuge tube filters prior to quantification. Drug quantification was performed using an Acquity™ Ultraperformance LC (UHPLC) coupled to a photodiode array (PDA) detector (Waters Corp, Milford, MA, USA). Samples were separated using an Acquity UPLC^®^ BEH C18 column (2.1 mm × 50 mm, 1.7 μm, Waters, USA) equilibrated at 40 °C. A 2-min gradient elution at a constant flow rate of 0.5 mL/min was performed using a mobile phase starting at 50% of a 0.05% (v/v) formic acid (Reactolab SA, Servion, Switzerland) aqueous solution and 50% of 0.05% (v/v) formic acid acetonitrile (Sigma-Aldrich, Saint Louis, MO, USA) solution. Wavelength detection was set at 262 nm on the photodiode array (PDA) detector. Peak due to itraconazole eluted at 1.27 min. Lower limit of quantification value was set according to signal/noise ratio > 6 and linearity R2 > 0.990 was obtained for the calibration curves ranging from 0.05 to 8 µg/mL. Statistical comparison between different groups was performed using One-Way analysis ANOVA (Tukey’s multiple comparison test).

## Formulations and drug delivery systems proposed for nanocrystals

### Nanosuspensions and tablets

Suspensions of nanocrystals (or nanosuspensions) represent the most common, simple, representative, and precursory formulation of this family (Fig. 1A). They can be directly obtained and even be practically ready to use via wet milling since the drug is generally suspended in an aqueous vehicle. The process of size reduction generates a thermodynamically unstable system by forming additional interfaces. These particles will tend to agglomerate to minimize their total energy, also known as Gibbs free energy [13]. Therefore, the incorporation of surfactants and/or stabilizers is needed to avoid this phenomenon as well as to improve the wetting properties of the particles in the vehicle [6, 13]. A recent research example in the field was reported by Paredes et al. in 2020 [14]. They aimed to develop poloxamer 188-stabilized ricobendazole nanocrystals by wet milling and spray-drying to treat helminthiasis. In this case, no dissolution rate improvement was observed in comparison with the physical mixture of ricobendazole and the poloxamer 188, used as a control. However, their redispersed nanocrystals showed an increased oral bioavailability in dogs. When compared to the micronized drug, a higher maximum plasma concentration (Cmax) and a 1.9-fold higher AUC_0−∞_ was observed. The authors suggested that the better in vivo performance of the nanocrystals was due to the higher specific surface area of the drug, which promotes higher saturation concentrations. Over the last decade, several studies on nanosuspensions have explored the oral route [15–17] but also others such as the ocular [18–21], nasal [22, 23], topical [24, 25], intra-articular [26], intra-muscular delivery [27] and even the inhalation route [28]. The cornea, for instance, might be permeable to nanosized drug particles. Based on this hypothesis, Baba et al. designed a fluorometholone nanosuspension-eye drop formulation to treat keratoconjunctivis [18]. They obtained rectangular-shaped nanocrystals around 200 nm that were stable for 6 months at 10 °C. Penetration and metabolization into the aqueous humor of rabbit eyes were compared with a micronized particle suspension. Results in rabbits showed that the ocular penetration of fluorometholone was 2- to 6-fold higher after 120 min.

Incorporating nanocrystals in tablets and capsules is the alternative classic strategy to aqueous nanosuspensions (Fig. 1B) [29–31]. In fact, the majority of nanocrystals in the market are nowadays formulated as tablets. As described in the last section of this article, they emerged in the early 2000s to improve the oral bioavailability of poorly water-soluble drugs [6, 31, 32]. However, there are only a few recently published studies on tablet formulations containing nanocrystals. In one such study, Naguib et al. developed sublingual tablets containing flibanserin nanocrystals to treat pre-menopausal hypoactive sexual desire disorders [30]. This drug is known to have poor aqueous solubility and therefore low oral bioavailability. Freeze-dried nanocrystals around 450 nm in size provided a saturated solubility five times higher than the pure drug. The optimized sublingual tablets, which disintegrated in about 36 s, were then tested in vivo in rabbits. Calculated pharmacokinetic parameters showed an enhanced bioavailability with a 2-fold AUC_0−∞_ increase. These outcomes were achieved due to the reduced particle size of flibanserin nanocrystals together with an acidic microenvironment generated in the presence of boric acid contained in the tablets.

Overall, these studies demonstrated the versatility of nanocrystals suspensions through their applications for different routes of administration. They can be easily formulated via a fast and straightforward method to prevent solubility issues. Tablets containing dried nanocrystals are a feasible option for oral administration. Despite the potential cost-effectiveness of tableting, the transformation into solid products requires a drying step, such as freeze-drying, spray-drying or granulation before compression [33]. These procedures often require the use of other excipients, such as cryo-protectants or matrix formers, which should be carefully selected to avoid potential interaction or interference with the nanocrystals during compression. Eventually, this can lead to a higher risk of irreversible aggregation, thus limiting the effective proportion of nanocrystals in tablets [34, 35]. This hinders the redispersion of the nanocrystals in the gastrointestinal tract and subsequent dissolution, reducing their advantages [10]. Nevertheless, a drying step is sometimes favorable to improve the stability of aqueous nanosuspensions [5]. This offers the possibility to concentrate the preparation and represent an intermediate formulation step, not only for tablets but also for other drug delivery systems shown in the following sections.

### Nanocrystal-loaded hydrogels

Hydrogels are cross-linked hydrophilic polymeric chains organized in a tridimensional matrix network embedding water. Nanocrystals can be easily embedded into these matrix scaffolds (Fig. 1C). Nanocrystals-containing hydrogels can be designed to improve specific characteristics such as bioadhesion, modulable viscosity, and even controlled release. This offers a high versatility and allows for topical administration on the skin, hair follicles, eyes, nasal cavity, and parenteral injections [36–38]. Together with faster drug dissolution rates and larger concentration gradients, drug diffusion through the tissue of interest can be increased *via* enhanced drug penetration and retention for an extended period [39–44]. Probably for these reasons, nanocrystals-containing hydrogels have become one of the most popular drug delivery systems reported in recent years. In one such study, an extended-release nanocrystal gel formulation was designed for dermal delivery to target hair follicles specifically [45]. Curcumin nanocrystals, chosen as a model drug, were incorporated in up to fourteen different gels with different viscosity and lipophilicity values. However, this did not influence nanocrystal penetration into hair follicles when tested in an ex vivo pig ear model. The authors suggested that the massage decreased the viscosity of the gels, sharing the same shear-thinning flow behavior. Still, embedding the nanocrystals into hydrogels facilitated application, adhesion on the tissue, and increased residence time of the formulation.

In situ forming hydrogels are currently gaining relevance in the field. They can be easily administered as a semi-solid/liquid form at room temperature, then gaining viscosity at body temperature to provide higher residence time or even specific functions such as joint lubrication/viscosupplementation. Tomić et al. intended to improve the efficacy and safety of topical acne treatment by formulating azelaic acid nanocrystals loaded in a hybrid poloxamer/hyaluronic acid in situ forming hydrogel [41]. This research group conducted a double-blind, randomized controlled study on patients with mild to moderate *acne vulgaris* comparing a 10% dug-loaded hydrogel with a commercial cream containing 20% o active pharmaceutical ingredient. Notwithstanding, their formulation showed better efficacy and safety after 8 weeks of daily treatment, with a significant reduction of acne-related inflammatory and non-inflammatory lesions. In another study, a higher docetaxel mucosal penetration was obtained by means of an in situ forming nanocrystal-hydrogel formulation for cervical cancer therapy [46]. For that, the surface of docetaxel nanocrystals was functionalized with the trans-activator of transcription (TAT) peptide, a common cell-penetrating peptide, using a polydopamine coating. The nanocrystals were further incorporated into a poloxamer 407-based thermosensitive gel. In vitro, TAT-coated nanocrystals per se showed higher cervical cancer cell uptake and growth inhibition compared to poly(ethylene glycol) (PEG)ylated nanocrystals. More importantly, an extended ex vivo and in vivo intravaginal retention on mice was observed with improved mucosal penetration and tumor growth inhibition when dispersed in the gel. Eventually, an injectable in situ forming hyaluronic acid hydrogel containing camptothecin nanocrystals was proposed by Yongsheng Gao and his team as a local and long-term delivery system for the treatment of rheumatoid arthritis [47]. The intra-articular injection into the joint of collagen-induced arthritis rats showed that the formulation was maintained for over four weeks. This correlated with the lowest levels of the inflammatory interleukin-1β after 60 days, compared to the controls.

All these studies demonstrate that hydrogels are potent topical or parenteral vehicles for nanocrystals. They provide a higher residence time and potentiate penetration into tissues, thus boosting drug bioavailability. Their semi-rigid polymeric network is thought to provide an extra nanocrystal physical stabilization meant to prevent re-crystallization issues. They can be easily manufactured by gelation of the aqueous nanosuspension or by simply redispersing the nanocrystals in a pre-formed gel. Hyaluronic acid is a privileged material but other biodegradable and biocompatible materials can be used for parenteral administration. In this regard, in situ forming hydrogels facilitate syringeability and injectability while providing viscosupplementation and a delivery depot on site.

### Microneedle-mediated delivery of nanocrystals

Topical application of nanocrystals has been proved efficient and relatively easy to formulate as seen in the previous section. However, the use of semi-solid pharmaceutical forms or patches still presents some limitations, mainly due to the low skin permeation and the need for frequent administrations [48, 49]. This led to the investigation of novel delivery systems designed to increase drug penetration and sustained release. Among them, microneedles have gained the attention of researchers as a result to their minimally invasive administration, high targeting and ability to incorporate poorly soluble drugs [50]. Microneedles, or microarray patches, are micron-sized spikes used to deliver active compounds transdermally (Fig. 1D). They can pierce and bypass the *stratum corneum* by forming micron-sized pores in the skin, directly delivering the drug into the dermal tissue [51]. In addition, drug-coated, hollow, and hydrogel-forming microneedles are composed of cross-linked polymers that can swell in contact with the interstitial fluid to form a drug reservoir on site. Dissolvable microneedles are composed of a biocompatible polymeric matrix, typically sugars, natural or synthetic polymers, in which hydrophobic drugs can easily be dispersed [49, 50, 52–55].

Vora and colleagues were in the first ones to incorporate vitamin D3 into dissolvable microneedles [56]. First, they used a sonoprecipitation method to obtain a nanosuspension. This nanosuspension was then mixed with a high molecular weight polyvinylpyrrolidone solution to obtain a gel, which was filled into a laser-engineered mold to form the microarrays. In vitro studies using Franz diffusion cells showed that the microneedles released significantly a 6.8-fold higher amount of vitamin D3 compared to the patches without microneedles. In another recent study, antiretroviral nanocrystals were loaded into a hollow microneedle delivery system

[57]. Nanocrystals of rilpivirine, a second-generation non-nucleoside reverse transcriptase inhibitor, and of the integrase inhibitor cabotegravir, were loaded into separate microneedle systems for the treatment of HIV (human immunodeficiency virus)-associated neurocognitive disorder. Both types of nanocrystals were obtained by wet milling, then lyophilized, reconstituted in water, and finally incorporated into 600 μm hollow microneedle arrays. The formulations were tested ex vivo on porcine skin and then administered to rats with an intradermal injection pad. Compared to the oral control administration, results indicated that both drugs were successfully delivered to the brain with higher AUC and C_max_ values after 3–4 weeks. Furthermore, rilpivirine delivery to the brain was therapeutically significant since the concentration of drug required for 90% inhibition of the reverse transcriptase (IC90) was reached after 4 weeks.

The above-mentioned studies exemplify the potential of combining nanocrystals with different types of microneedle delivery systems. Although this field has been scarcely explored so far, these formulations can improve the transdermal delivery of active compounds at high doses. In addition, other routes of administration could benefit from microneedle systems containing nanocrystals to improve both drug penetration and sustained release. In fact, recent investigations in microneedle-mediated minimally invasive intra-ocular delivery have shown promising outcomes [58]. This technology is also expected to improve therapeutic efficacy and patient compliance. Even though the scalability potential remains unclear, this technology might enable the delivery of poorly soluble drugs to tissues that are unreachable with classic topical formulations.

### Nanocrystal-polymer microparticles

Patients suffering from chronic diseases often need to follow a treatment for life or an extended period. Compliance may be partial, which affects clinical outcomes. The integration of nanocrystals in polymeric hydrophobic microparticles or microspheres (Fig. 1E) can provide an additional biodegradable solid shell that modulates and prolongs the release kinetics [59–61]. This can yield also an injectable formulation that confers a long-term drug/nanocrystal release on site, thus reducing the frequency of administrations.

**Fig. 2:**
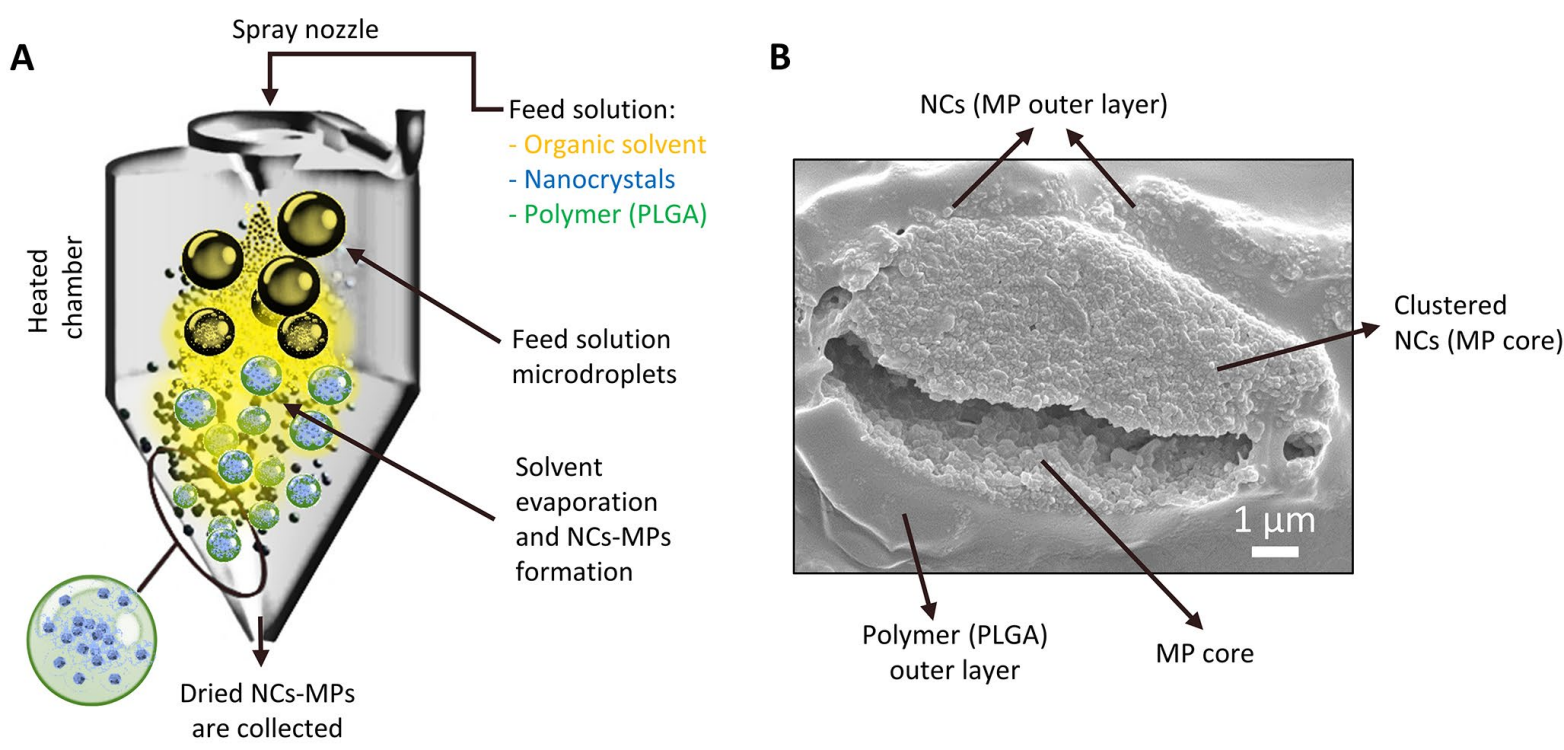
(A) Schematic representation of a spray-dryer and the formulation of nanocrystals (NCs) loaded in polymeric microparticles (MPs). Briefly, the feed solution containing the polymer poly(lactic-co-glycolic acid) (PLGA) dissolved in an organic solvent together with nanocrystals in suspension is sprayed into a pre-heated chamber. Solvent evaporation from droplets leads to NC-MP formation and collection. (B) Scanning electron microscopy representative micrograph of a NC-MP cross section under investigation by our research group. Magnification is x10,000. For further details about the formulation approach and sample preparation visit [64]

Progressive cartilage degeneration and chronic inflammation are two factors associated with knee osteoarthritis. Current treatments are inefficient or require frequent intra-articular injections of drugs due in part to fast clearance in the joint space [62]. Based on this premise, our group proposed to combine the properties of hydrophobic polymer microparticles with nanocrystal technology to obtain high drug-loaded formulations for long-term local release. In one paper by Maudens et al. kartogenin, a very poorly soluble drug that promotes articular cartilage regeneration, was wet-milled [60]. The obtained nanocrystals of 320 nm were then freeze-dried and embedded in poly (DL-lactide) microparticles of 10–20 μm by spray-drying. The formulation showed an extended-release profile with 62% of katogenin released over 3 months. In vivo studies in osteoarthritic mice showed higher cartilage regeneration activity compared to free kartogenin. In a similar study, celecoxib nanocrystals were embedded in poly (DL-lactide) microparticles to treat chronic inflammation associated with osteoarthritis [63]. Noteworthy, a very high drug loading of 50% w/w with an encapsulation efficiency above 80% was obtaied for a poorly soluble drug (i.e., celecoxib). Although high drug payloads usually correlate with fast release, in this case, an extended biphasic in vitro drug release over 3 months was observed. Drug loading in this formulation approach is not limited by the solubility of the drug in the spray-drying feed solution since the drug is majorly loaded as nanosuspension. Extended drug release over several months was highly influenced by the solubility and specific surface area of the nanocrystals, rather than by the microparticle polymer type, which is the case when a drug is dissolved in a matrix. The formulation procedure shown in Fig. 2A was recently implemented for the encapsulation of the GLPG0555 in collaboration with Galapagos NV (Mechelen, Belgium) [64]. A representative cross section micrograph showing the internal structure of this type of particles is also shown in Fig. 2B.

This combined technology (wet milling + spray-drying) enabled encapsulating nanocrystals into microparticulate systems for the first time for parenteral (e.g., intra-articular) administration. The two processes are scalable and can be exploited to encapsulate a large payload of a non-soluble drug into a sustained-release formulation. In addition, the spray-drying technique allows tailoring the properties of the resuspendable and injectable powder. For instance, a particle size around 10–20 μm is thought to slow down the clearance or filtration of the drug nanocrystals towards the systemic circulation compartment. Taken together, these methods can lower injection volume, which is crucial for non-intravenous (IV) parenteral administration. For those reasons, this technological platform should be explored in the future for other non-IV parenteral administrations such as intraocular delivery.

### Liposomal and surface-engineered nanocrystals

The progress of nanotechnology in recent years has also provided the possibility of engineering drug nanocrystals to ameliorate their therapeutic index. This can be crucial in diseases where systemic exposure of drugs is often associated with serious adverse effects, such is the case of cancer therapy [65]. Some studies have recently investigated the incorporation of nanocrystals into liposomes (Fig. 1F). Drug nanocrystals can be incorporated or formed in situ within the lipid layers [66–68]. Li et al. developed in situ forming nanocrystals of ciprofloxacin in liposomes for oral delivery [69]. In this case, the drug precipitates inside the vesicles following a freeze-thawing step. The release profile of the liposomes in vitro was dictated by the solid state of the drug with a steady release behavior under non-digestive conditions that increased afterward under a simulated intestinal fluid medium. The surface of liposomes can also be functionalized to provide a specific ligand-mediated targeting effect [70, 71]. Alternatively, a two-step method was proposed for a targeted liposomal delivery system model for hydrophobic antitumoral drugs in another study [72]. Nanocrystals were first obtained by wet ball-milling technique and then incorporated into PEGylated and folic acid-functionalized liposomes. These liposomes displayed enhanced colloidal stability with a drug loading of up to 20%. In vivo studies in K56 xenograft mice showed higher tumor targeting/accumulation after parenteral administration in comparison with either the free nanocrystals or the non-targeted liposomes.

Coating or decoration of nanocrystals is gaining relevance, particularly in the field of cancer therapy and bioimaging. One of these approaches consists of grafting the nanocrystals’ surface with diverse compounds such as proteins or PEG. This usually aims at improving drug bioavailability and biocompatibility after IV injection, by reducing non-specific protein adsorption and phagocytosis [73–75]. Park et al. published in 2017 a study using albumin-coated nanocrystals of paclitaxel to improve the therapeutic outcomes for solid tumors [76]. These authors formulated paclitaxel nanocrystals by crystallization in various surfactant-containing mediums. The formulation stabilized with poloxamer 407 displayed the smallest size and a rod-shaped morphology thought to elude macrophage uptake. Then, nanocrystal surface coating was performed by incubating the nanocrystals with albumin for 24 h at room temperature, prior to centrifugation to remove the excess of unadsorbed protein. In comparison with Abraxane^®^, the commercial albumin-bound paclitaxel, at the equivalent dose of 15 mg/kg, this formulation showed a higher antitumor efficacy when tested in a mice model bearing subcutaneous melanoma. These results correlated with higher paclitaxel tumor accumulation (27.4 µg/g vs. 13.8 µg/g for Abraxane^®^). In a recent study, polydopamine coated paclitaxel-PEG nanocrystals were dispersed in electrospun nanofibers. This innovative nanocrystalline-in-nanofiber implant device showed improved anti-tumor efficacy in a murine cervicovaginal tumor model together with a prolonged vaginal residence, higher transmucus penetration and minimal mucosal irritation [77].

Nanocrystals were not initially specifically designed to increase drug targeting or have stealth properties via surface engineering. However, the concomitant progress of nanotechnology allows nowadays to confer these characteristics to nanocrystals, owing to their own versatility and workability. Although adding manufacturing complexity, the development of nanocrystals in liposomes might also solve some of the stability and biocompatibility constraints reported/observed in classical nanocrystal formulations. In that sense, elucidating their localization, physical state inside the liposomes is encouraged [78]. For instance, the well-known anticancer nanomedicine Doxil^®^ is reported to display stabilized recrystallized doxorubicin inside the liposomes [79]. All these upgrades might prompt the consolidation of safe and effective parenteral administrations of nanocrystals, and this can be also applied to others such is the case of the ocular delivery [80]. With similar goals as for liposomal formulations, coating of nanocrystals aimed to increase drug bioavailability holds promise due to the simplicity of the formulation process. However, further in vivo studies are still required to ratify the benefits of this surface decoration strategy.

## Case study: development of an itraconazole nanosuspension

The present paper includes an informative practical case. It is meant to exemplify the simplicity, versatility and technicalities of the approaches described in the previous section. In this context, we aimed to improve the therapeutic perspectives of the poorly-water soluble drug itraconazole by preparing a nanosuspension and a hydrogel. Itraconazole is a model drug for development of small molecule formulations because of its extremely low aqueous solublity (1–4 ng/mL) [81]. This weak base (pKa = 3.7) molecule with a logP of 5.6 belongs to a class of drugs known as azole antifungals. For that reason, the marketed oral solution Sporanox^®^ solubilizes it in a concentrated cyclodextrin solution at pH 2 [82]. In the following case study, we aimed to formulate a nanosuspension by a top-down approach to overcome the limited solubility of this drug.

**Fig. 3:**
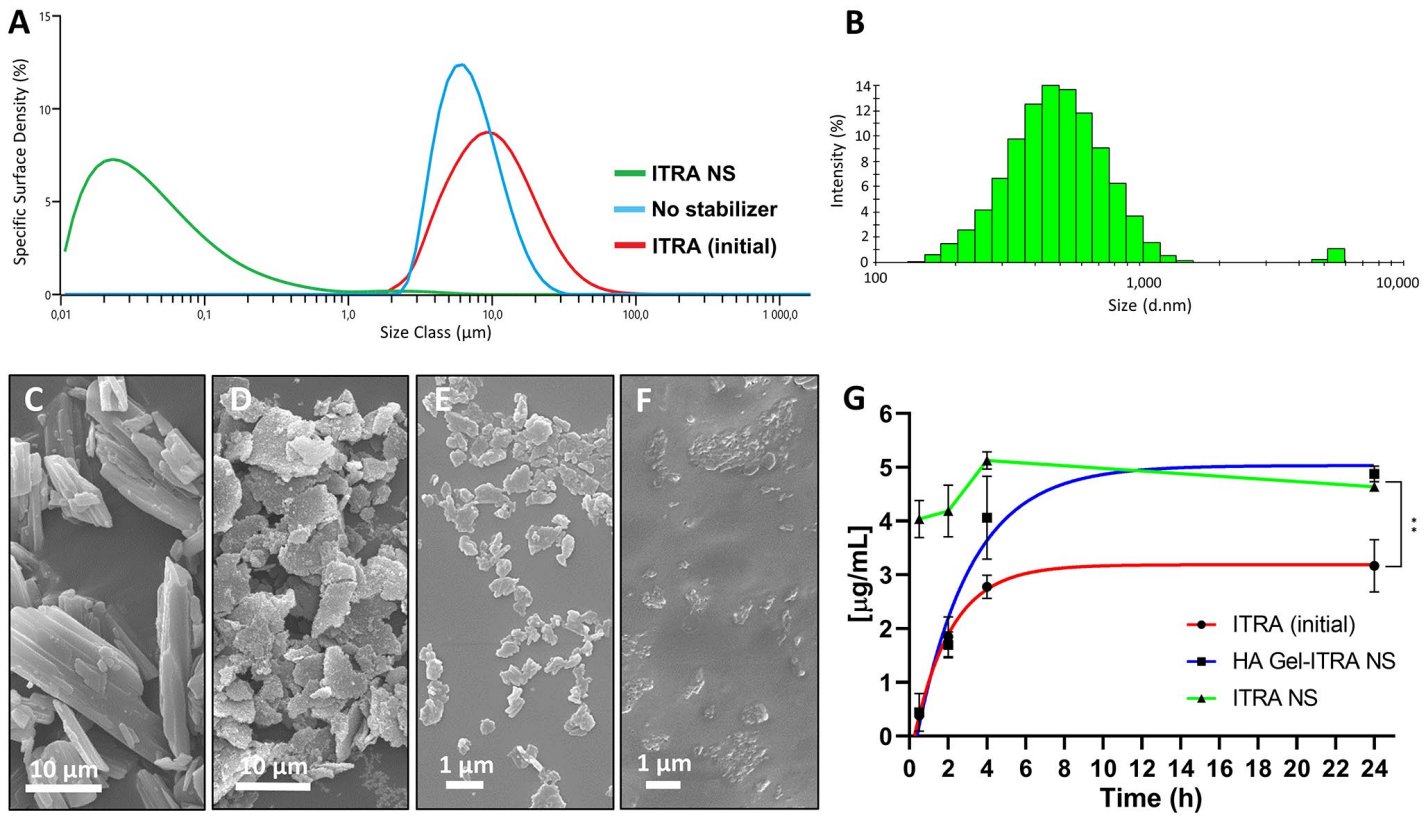
Characterization of wet-milled itraconazole (ITRA). (A) Particle size distribution representative graphs by specific surface density obtained by laser diffraction. (B) Particle population distribution by intensity representative peak curve of ITRA nanosuspension (NS) measured by dynamic light scattering. Representative scanning electron micrographs of (C) initial ITRA, (D) wet-milled ITRA without stabilizer, (E) poloxamer 407 (P407)-stabilized ITRA NS and (F) P407-stabilized ITRA NS dispersed in a hyaluronic acid (HA) gel. (G) Dissolution profile of ITRA NS (green), HA Gel-ITRA NS (blue), and initial ITRA (+ P407) (red). Blue and red curves were fitted according to a pseudo first-order release kinetics. All results are represented by the mean ± sd (*n* = 3). ** *p* < 0.01

Wet milling was performed based on previous experience to obtain a main particle size distribution in the nanosize range (for further information see Sect. 2.2). Laser diffraction results in Fig. 3A indicated that nanosize reduction of itraconazole (Dv50: 0.52 μm) succeeded in the presence of stabilizers (i.e., poloxamer 407), in agreement with previous reports [6, 76]. After milling, the specific surface area values increased by 100-fold in comparison with the initial drug (50,610 vs. 445 m^2^/Kg, respectively) as clearly depicted in Fig. 3A. Practically, dynamic light scattering results showed that this itraconazole nanosuspension exhibited a heterogeneous population of particles with a mean hydrodynamic diameter size and polydispersity index of around 630 nm and 0.483, respectively (Fig. 3B). Scanning electron microscopy confirmed the size reduction of the initial material shown in Fig. 3C. We could observe that in the absence of a stabilizer and even though particles showed certain attrition, they clearly presented agglomeration (Fig. 3D). On the contrary, the main population of the wet-milled poloxamer 407-stabilized itraconazole particles in Fig. 3E were below 1 μm, in agreement with the other techniques. Finally, dissolution studies demonstrated that the itraconazole nanosuspension (Fig. 3G, in green) dramatically increased the dissolution rate profile of the pure drug (Fig. 3G, in red) from the beginning of the experiment. Interestingly, when the nanosuspension was loaded in a hyaluronic acid hydrogel (Fig. 3F), we were able to tailor the drug release profile of the formulation. For instance, the burst release of the native nanosuspension was no longer observed (Fig. 3G, in blue). Finally, itraconazole concentrations quantified in solution at 24 h for the two nanosuspension formulations were significantly higher in comparison with the initial compound (4.6/4.7 vs. 3.1 µg/mL, respectively).

The present case study illustrates how *via* wet milling, a simple and scalable manufacturing process [11], an itraconazole nanosuspension formulation with improved characteristics was successfully developed. In agreement with the content in Sect. 3, we have demonstrated that the higher specific surface area of the nanosuspension translates into higher dissolution rates and solubility. This is again consistent with the the Noyes-Whitney and Ostwald–Freundlich Eqs. [7, 8], and the results were also in line with the itraconazole solubility values reported for this setup [82, 83]. It is essential to keep in mind that this ultra-fine particle size also enables the possibility of a direct parenteral injection [84, 85]. As seen in Sect. 3.2, it was also easy to embed this nanosuspension in a biodegradable and biocompatible hydrogel matrix (hyaluronic acid) in order to control their release profile. This reinforces the versatility and feasibility of the approach and stands for a realistic alternative in the drug delivery field for poorly soluble drugs in the pipeline of pharmaceutical industries.

## Historical and present panorama

**Fig. 4:**
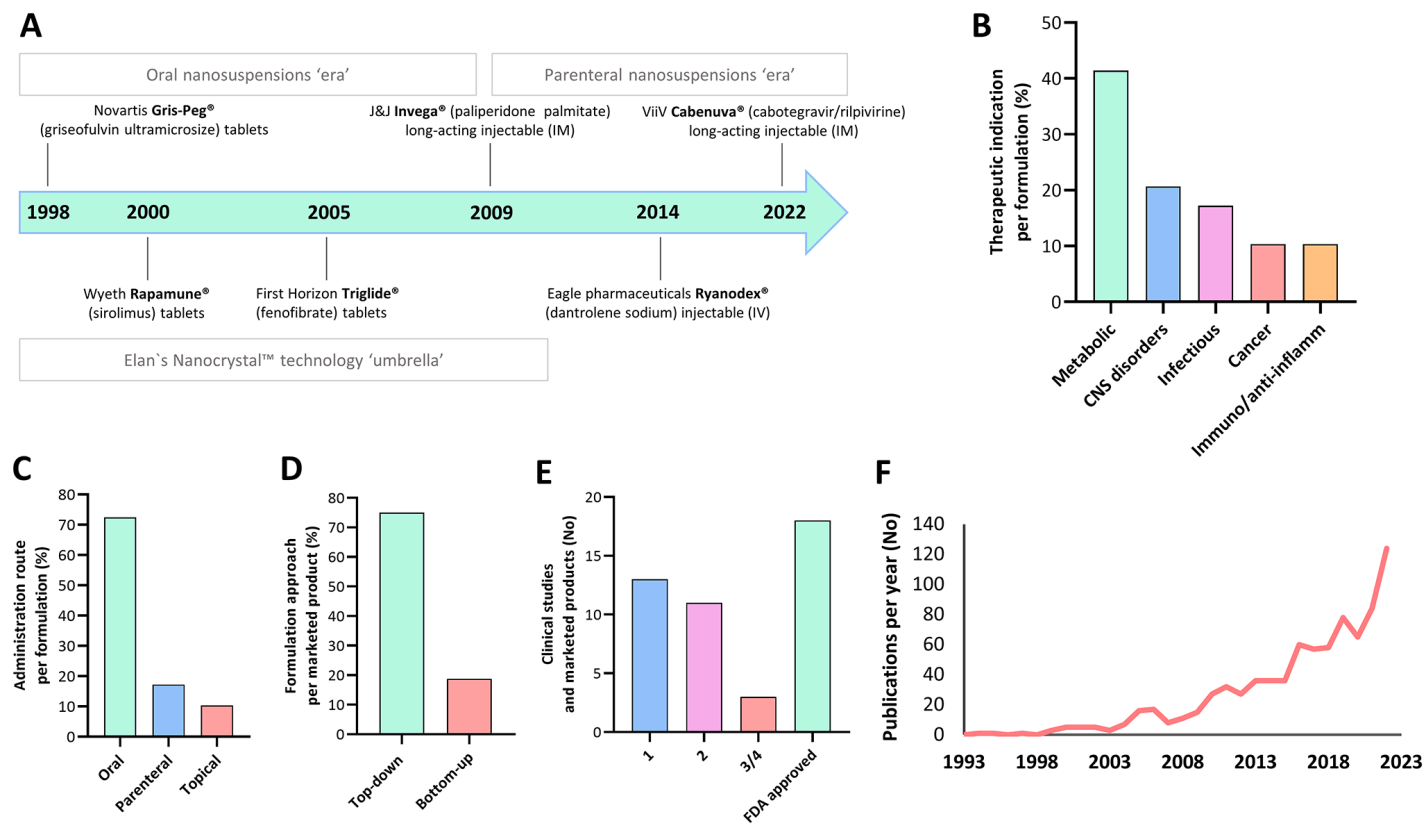
Nanocrystal formulations: a state-of-the-art estimation, based on an October 2023 search on Clinicaltrials.gov and MEDLINE databases. For further details on methodology, see Sect. 2.1. (A) Historical timeline in the field of marketed nanocrystal and nanosuspension formulations. (B) Therapeutic indications (diseases) by percentage correspond to the formulations found in clinical trials and on the market. (C) Administration routes by percentage correspond to the formulations in clinical trials and approved. (D) Formulation approaches by percentage correspond to the approved formulations and in clinical trials. (E) Number of studies in clinical phase trials and formulations in the market in 2023. (F) Publications per year in the field of drug nanocrystal and nanosuspension formulations

At the beginning of Sect. 3, we commented on the advent of nanocrystal technology in the clinic as an efficient but also lucrative tool to address the problems of poorly-aqueous soluble drugs. Looking at the historical timeline in Fig. 4A, pioneer ultramicrosized suspensions began to appear in the late 90s. Just after, in 2000, the FDA approved the first ‘official’ nanocrystal-tablet formulation Rapamune^®^ (sirolimus) [86]. This marked the ‘bloom’ of nanocrystal formulations for oral administration as seen in Table 1. In the following five years, up to nine products (half of the available repertoire) were licensed under the Elan’s nanocrystal^®^ technology umbrella with different partners [32, 86]. Among them, Emend^®^ (aprepitant), Triglide^®^ (fenofibrate), or the previously mentioned Rapamune^®^ reached the market as tablets or capsules during these years. Figure 4B shows that the majority (40%) of formulations on the market and clinical trials are intended for the treatment of metabolic diseases (hypertension, lipid disorders, etc). This may partially explain why the oral route prevails here, as seen in Fig. 4C. We had to wait until the end of the first decade of the 21st century to see the first long-acting nanocrystal injectable formulation, Invega^®^ (paliperidone palmitate). An interesting attribute of this formulation platform is that the different particle size and concentration allows for modulation of the controlled release profile from 1 (Sustenna) to 4 months (Trinza), approximately [87]. This could have paved the way for the market access of new nanosuspensions in the following years, but the expectations have surprisingly cooled down to date. One solely but recent success is the approval of Cabenuva^®^, the first long-acting antiretroviral injectable nanoformulation composed of cabotegravir and rilpivirine. This once-monthly injected drug combo nanoformulation is showing promising results in maintaining HIV-1 suppression in patients [88].

To better appreciate the reasons for this abrupt halt we should have in mind some insights. In the first place, Elan’s nanocrystal^®^ technology is estimated to be protected by more than 100 patents worldwide [89]. This might not have been affordable for small/middle-sized companies or attractive enough for the big pharmaceutical holdings, with a large number of soluble drugs still in their pipeline. In the same period, the approval in 2002 of the ‘blockbuster’ Humira^®^ (adalimumab) marked the consolidation of biologics at the expense of small molecules [90]. The investment context did not improve with the economic crisis of 2008, precisely when one of the last nanocrystal formulations (i.e., Invega^®^ in 2009) was approved. However, more importantly, the emergence of the so-called amorphous solid dispersions, along with the advances in spray-drying and hot melting extrusion came out on top for the efficient tableting of poorly water-soluble drugs [91]. In recent years, the Covid-19 crisis diverted efforts towards the development of the tandem gene therapy and lipid nanoparticles. Concerning regulatory compliance, this should be in any case beneficial for nanomedicines in the future, and that should also include nanocrystals. Finally, whether we link it or not to the high preclinical and clinical failure rate of innovative drugs, especially non-soluble, we must inexorably recount the limitations of this kind of formulations. Operational, long-term, and physiological stability, including crystal growth and sedimentation, is one of the main hurdles hampering safety and handling, but also efficacy [92]. In this line, formulation scientists often show, as we did with the case study presented in Sect. 4, faster dissolution rate profiles in artificial media or in phosphate-buffered saline solutions in the best-case scenario. This may not always be sufficient to reach therapeutic concentrations over time in vivo. The “Spring Parachute” dissolution model can also explain this event [93]. Here, drug peak concentrations in solution (see Fig. 3G, in green) are progressively reduced over time as the stabilizer leaves the nanocrystal surface. Regarding manufacturing, top-down approaches mainly entailing wet milling and high-pressure homogenization are the most popular (Fig. 4D). This is probably because they are easily scalable, show low batch-to-batch variation, and do not require organic solvents [92]. Nevertheless, there is still a risk to consider, specifically the contamination of the nanocrystals by metals, ceramic materials, or other milling machine debris. This needs to be carefully assessed especially in the case of parenteral administration [94].

**Table 1:** Nanocrystal and nanosuspension formulations in the market

Trademark (drug– Company)	Dosage form	Indication	Year of approval
Verelan PM^®^ (verapamil– Schwarz Pharma)	Tablet	Hipertension	1998
Gris-Peg^®^ (griseofulvin– Novartis)	Tablet	Fungal infection	1998
Azopt^®^ (brinzolamide– Alcon)	Ophthalmic suspension	Glaucoma	1998
Rapamune^®^ (sirolimus– Wyeth)	Tablet	Immunosuppression	1999
Avinza^®^ (morphine sulfate– King Pharma)	Capsule	Chronic pain	2002
Ritalin LA^®^ (methylphenidate– Novartis)	Capsule	Behavioural disorders	2002
Zanaflex^®^ (tizanidine– Acorda)	Capsules	Spasticity management	2002
Herbesser^®^ (diltiazem– Mitsubishi Tanabe)	Tablet	Hipertension	2002
Emend^®^ (aprepitant– Merck)	Capsule	Nausea (chemotherapy)	2003
TriCor^®^ (fenofibrate– Abbott)	Tablet	Hypercholesterolemia	2004
Focalin XR^®^ (dexmethylphenidate– Novartis)	Capsule	Attention deficit disorder	2005
Triglide^®^ (fenofibrate– First Horizon)	Tablet	Lipid disorders	2005
Megace ES^®^ (megestrol acetate– Par Pharmaceutical)	Oral suspension	Anorexy	2005
Naprelan^®^ (naproxen– Pfizer/Wyeth)	Tablet	Analgesia	2006
Cesamet^®^ (nabilone– Lilly)	Capsule	Nausea (chemotherapy)	2006
Invega^®^ (paliperidone palmitate– Johnson & Johnson)	Tablets/injection (i.m.)	Schizophrenia	2006/2009
Ryanodex^®^ (dantrolene Sodium– Eagle Pharmaceuticals)	Injection (i.v.)	Malignant hyperthermia	2014
Cabenuva^®^ (cabotegravir/rilpivirine– ViiV Healthcare)	Injection (i.m.)	HIV-1 infection	2022

HIV-1, human immunodeficiency virus. *Some authors include also hydroxyapatite nanocrystals and paclitaxel-albumin nanoparticles. Updated from [32, 86, 92]

In spite of the above-mentioned challenges, it still draws our attention that there is a lack of ongoing Phase III clinical trials (Fig. 4E). We only found two studies corresponding to a nasal spray ivermectin nanosuspension for Covid-19 management (NCT04951362). Moreover, many of the studies in Phase II correspond to different anticancer indications of the Panzem^®^ (2-methoxyestradiol) nanocrystal colloid dispersion. While this is underwhelming, it is convenient to bear in mind that there are around twenty nanocrystal or nanosuspension formulations on the market in 2023 (Fig. 4E; Table 1). This represents around the 25–30% of the total nanomedicine market [95]. It is not negligible, considering that the global nanotechnology market size reached USD 210 billion in 2022, and is expected to double over the next 10 years [96]. The historical timeline in Fig. 4A suggests that these marketed nanocrystals were ahead of their time as well. Precisely when the so-called ‘golden era’ of nanocrystals was practically over around 2009, publications on the topic started to grow progressively as depicted in Fig. 4F. The approval of the first liposomal nanomedicine Doxil^®^ (liposomal doxorubicin) in 1995 took around 30 years since the first liposomal structures were reported [97]. Yet, time is needed to confirm if this curve will continue to grow exponentially or is just a fleeting trend. This means that nanotechnologists are nowadays actively integrating nanocrystals in their novel drug delivery systems or giving them new roles as summarized in Sect. 3; but why now? Among other reasons, because this technology is relatively simple, versatile and because 90% of the current drug pipeline faces solubility issues [98]. Drug loadings can be also very high (close to 100%), resulting in more drug concentration in less volume. A large part of the success of the anticancer nanomedicine Abraxane^®^, considered also as a nanosuspension by some scientists, relies on being just paclitaxel bound to albumin [32].

Still, we have to acknowledge that there is significant work ahead of us. For instance, formulators should explore innovative ways to control nanocrystal redispersibility after tableting. It is essential to understand better how nanocrystals permeate and penetrate subcutaneous tissues and biological barriers when applied topically. In that sense, the incorporation of the above-mentioned cabotegravir/rilpivirine nanocrystals (Cabenuva^®^) in microneedles might further ameliorate the therapeutic outcomes and patient compliance in HIV patients. In addition, nanocrystal cell internalization and drug dissolution profiles should be thoroughly evaluated in vitro/ex vivo using biological fluids. The field of parenteral injections offers ample opportunities for exploration and growth, and this not only includes the intramuscular route but also others such as the intra-vitreal or intra-articular routes. Attention should also be given to the particle size, shape (i.e., irregular and not spherical) and crystalline state of nanocrystals. All these can affect sedimentation, crystal growth, and interaction with cells, proteins and tissues, altering especially their physiological solubility and clearance/filtration towards other compartments. As highlighted in Sect. 3, formulations at the cutting edge such as liposomes, in situ forming hydrogels, or long-acting polymeric microparticles loaded with nanocrystals are promising candidates for this application (Fig. 1CEF). In addition, most of these studies report the implementation of polymers, lipids, surfactants and other excipients already approved by regulatory agencies (e.g., poly (DL-lactide), hyaluronic acid, PEG and poloxamers), which indeed facilitates translational research. The increasing number of studies shown in Fig. 4F may bridge the current technological gap from the bench to the clinic in the near future as well. This incoming generation of nanocrystal formulations will likely be integrated as a component in future drug delivery systems.

## Data Availability

The data presented in this study are available on request from the corresponding author.
